# Exploring the factors and personality traits influencing neurosurgery career choice in Jordan

**DOI:** 10.1007/s00423-026-04068-5

**Published:** 2026-05-12

**Authors:** Mohammed Nawaiseh, Tasnim Ghassab Alsaqer, Dana Y. Tarawneh, Haneen Khaled Diab, Qais Nawaiseh, Ashraf Nayef Alsawareah, Mohammad Aladawi, Ibrahim Abuelbeh, Vincent Q. Sier, Joost R. Van Der Vorst, Fawaz Al-Mufti

**Affiliations:** 1 Department of Ophthalmology, Jordanian Royal Medical services, Amman, Jordan; 2 Department of Neurosurgery, Faculty of Medicine, Hashemite University, Zarqa, Jordan; 3 School of Medicine, The University of Jordan, Amman, Jordan; 4 Department of General Surgery, Queen Alia Military Hospital, Amman, Jordan; 5 Department of Neurology, University of Missouri, Colombia, MO USA; 6 BlueSky Neurosciences, CarePoint Health, Kansas City, MO USA; 7 Department of Urology, Manchester Royal Infirmary, Manchester, UK; 8 Department of Surgery, Leiden University Medical Centre, Leiden, The Netherlands; 9 Departments of Neurology and Neurosurgery, Westchester Medical Center at New York Medical College, Valhalla, NY USA

**Keywords:** Neurosurgery, BFI-2, personality traits, Factors, Career choice, Future specialty

## Abstract

**Purpose:**

It is of common knowledge that choosing a medical specialty is a crucial career-defining decision. In this study, we investigated the relationship between personality traits, influencing factors, and the preference for neurosurgery among medical students and recent graduates in Jordan.

**Methods:**

A cross-sectional study was conducted using a web-based survey that was distributed to fifth- and sixth-year medical students and interns across five Jordanian universities. Descriptive statistics, chi-square tests, t-tests, and binary logistic regression were employed to identify associations between specialty choice and relevant factors.

**Results:**

We found that 5.2% (*n* = 53 out of 1012) of participants were interested in neurosurgery. Key predictors of neurosurgery choice included high GPA influence (Odds Ratio [OR] = 2.45, *p* = 0.004), intellectual challenge (OR = 2.23, *p* = 0.021), and open-mindedness (OR = 2.61, *p* = 0.002). Being a female (OR = 0.43, *p* = 0.005) and valuing lifestyle flexibility (OR = 0.48, *p* = 0.045) were associated with lower odds of choosing neurosurgery.

**Conclusion:**

Certain personality traits along with high GPA influence and intellectual challenge significantly influence the selection of neurosurgery. Concerns about lifestyle balance and gender disparities prevented many from choosing this demanding specialty.

**Supplementary Information:**

The online version contains supplementary material available at 10.1007/s00423-026-04068-5.

## Introduction

Choosing the appropriate medical specialty marks a major turning point for medical students and is not just a matter of personal preference. Their choice not only dictates future academic and career prospects, but it also impacts career satisfaction, personal life, and, most importantly, quality of patient care. A mismatch between a physician’s capacity and the demands of their chosen specialty might lead to a higher degree of job dissatisfaction, burnout, and eventually, compromised patient outcomes [1]. 

There exists an apparent reduced interest in pursuing a career in neurosurgery among medical students and young physicians. This declining interest is largely attributed to the stigma and negative perceptions surrounding the specialty, as reported in various regions, including Africa, where 76% of respondents acknowledged a shortage of neurosurgeons. Neurosurgery is regarded as one of the most challenging medical specialties, marked by poor work-life balance, lengthy residency training, long operating hours, perceived negative impact on family and social life, and significant physical and psychological demands coupled with intense shifts. Additionally, inadequate exposure to neurosurgery during medical school, especially at institutions without dedicated neurosurgery programs, further contributes to the limited appeal of the specialty [2–13]. 

On the contrary, previous studies have suggested that factors such as early exposure to the field, mentorship, intellectual stimulation, significant patient impact, perceived prestige, and personal interest may positively influence the choice of neurosurgery as the future chosen specialty [2, 3, 7–9]. Furthermore, personality is a central factor in the behavior and choices of individuals and has been associated with career preferences and satisfaction. Current studies on the personality of medical professionals, and neurosurgery in particular, have been largely underexplored in the Middle East [14]. Given the global and regional shortage of neurosurgeons, understanding these determinants is crucial for developing strategies to increase interest in the field and to ensure an adequate future workforce.

Aim.

In this study, we explore the relationship between personality traits, personal factors, and the preference for neurosurgery as the chosen future specialty among medical students and postgraduate interns across all Jordanian universities.

## Materials and methods

### Study design and participants

This cross-sectional study included fifth- and sixth-year medical students, and immediate internship postgraduate doctors from five Jordanian universities: The University of Jordan, Jordan University of Science and Technology, University of Mutah, Yarmouk University, and Hashemite University. The study targeted 5252 medical students and new graduates, and 1012 completed the questionnaire. Data was collected from participants by convenience sampling using a web-based survey service. A well-designed survey has been sent to all fifth- and sixth-year medical students and internship postgraduate doctors at each university using official university online Facebook groups. Explicit permission was obtained from participants by signing a statement of agreement at the start of each questionnaire.

### Data collection and study tools

Data collection took place between March and April 2020, utilizing the Big Five Inventory-2 (BFI-2) to assess the personality traits of each participant. The BFI-2 is a reliable and precise personality assessment that has been validated through multiple studies [15, 16]. However, the BFI-2, like other personality assessments, has limitations due to self-reporting. The BFI-2 was administered in English, which is the teaching language in all Jordanian medical schools. An Arabic translated BFI-2 test was not used as no validated version of the test was available at the time of the study. Participants were informed that they could contact the researchers, who are proficient in both English and Arabic, at any time for clarification regarding the BFI-2 content or any other part of the questionnaire. This was intended to reduce potential language-based misunderstandings. We gathered participant demographics such as age, gender, university, academic year, nationality, and location of permanent residence.

The study surveyed students about their preferred specialty, number of specialties of interest and their preference for practicing or non-practicing medical doctors. Practicing doctors had direct patient interaction, while non-practicing doctors worked in administrative positions. Those who choose neurosurgery were selected for this study for further analysis of the collected data [17]. 

Participants were questioned about the factors which influence their choice of a future medical specialty. The responses were on a 5-point Likert scale, ranging from strongly agree to strongly disagree.

### Ethical consideration

Jordan University Hospital’s IRB committee approved the study and participation was voluntary. Participants had the choice to withdraw at any moment. Participants’ names were not included in the study questionnaire to preserve their privacy. Data was kept completely private and anonymized using codes. Informed consent was obtained from all individual participants included in the study.

### Statistical analysis

Descriptive statistics were used to summarize the general characteristics of the study participants, including gender, age, and chosen specialty, as presented in Table 1. To assess the factors influencing students’ choice of neurosurgery as a future specialty compared to students who chose other specialties, a chi square test was conducted, as outlined in Table 2. Additionally, a t-test was conducted to compare personality traits between students who chose neurosurgery vs. other specialties, with the results presented in Table 3. Finally, binary logistic regression (Table 4) was employed to assess factors influencing specialty choice as neurosurgery. Variables that demonstrated statistical significance (p < 0.10) in previous analyses (e.g., chi-square and t-tests) were entered into the regression model. A significance level of p < 0.05 was considered statistically significant in the regression analysis. To facilitate the Chi-square and regression analyses, the 5-point Likert scale was dichotomized. Responses of ‘strongly agree’ and ‘agree’ were collapsed into an ‘agree group,’ while ‘neutral,’ ‘disagree,’ and ‘strongly disagree’ were collapsed into a ‘disagree group’. Finally, we have conducted a gender-stratified analysis, to assess the effect of gender on the influencing factors using chi-square (Appendix 1) and on personality traits using t-test (Appendix 2).

**Table 1:** General characteristics of the study participants

	Level	*N* (%) *
Gender	Male	398 (39.3%)
	Female	614 (60.7%)
Age (Years)		23.3 ± 1.2
Specialty	Neurosurgery	53 (5.2%)

*Mean ± Standard deviation for age

**Table 2:** Differences between those aspiring neurosurgery and those aspiring other specialties regarding factors influencing students’ choice of future specialty

Factor	Level	Other (*N* = 959)	Neurosurgery (*N* = 53)	*P*-value
Anticipated income	Agree*	626 (65.3%)	32 (60.4%)	0.467
Lifestyle or flexible work schedule	Agree	708 (73.8%)	27 (50.9%)	0.000
Workload or working hours	Agree	598 (62.4%)	21 (39.6%)	0.001
Absence of emergencies	Agree	412 (43.0%)	13 (24.5%)	0.008
Professional prestige	Agree	512 (53.4%)	30 (56.6%)	0.648
Specialty is well-respected	Agree	603 (62.9%)	36 (67.9%)	0.458
Occupational satisfaction	Agree	824 (85.9%)	47 (88.7%)	0.573
Personal interests	Agree	855 (89.2%)	46 (86.8%)	0.592
Academic interests	Agree	673 (70.2%)	37 (69.8%)	0.955
Personal skills or competencies in the specialty	Agree	753 (78.5%)	41 (77.4%)	0.841
Influence of media	Agree	217 (22.6%)	15 (28.3%)	0.339
Influence of family members	Agree	294 (30.7%)	19 (35.8%)	0.426
Influence of a professor or resident during medical school	Agree	420 (43.8%)	24 (45.3%)	0.832
Role model	Agree	365 (38.1%)	21 (39.6%)	0.820
Influence of having a doctor in the family	Agree	220 (22.9%)	17 (32.1%)	0.126
Influence of GPA	Agree	205 (21.4%)	20 (37.7%)	0.005
Career opportunities (availability)	Agree	585 (61.0%)	31 (58.5%)	0.715
Desire to specialize abroad	Agree	552 (57.6%)	36 (67.9%)	0.137
Intellectual challenge	Agree	514 (53.6%)	41 (77.4%)	0.001
Long-term relationship with patients	Agree	365 (38.1%)	18 (34.0%)	0.549
Gender distribution in the specialty	Agree	241 (25.1%)	14 (26.4%)	0.834
Peer group choices	Agree	134 (14.0%)	9 (17.0%)	0.541
Personal experience with a disease	Agree	281 (29.3%)	15 (28.3%)	0.876
Diversity of patients	Agree	392 (40.9%)	25 (47.2%)	0.365
Lack of competitiveness	Agree	312 (32.5%)	15 (28.3%)	0.521
Length of training	Agree	345 (36.0%)	12 (22.6%)	0.048
Gender	Male	367 (38.3%)	31 (58.5%)	0.003
	Female	592 (61.7%)	22 (41.5%)	
Academic year	Fifth	390 (40.7%)	23 (43.4%)	0.859
	Sixth	343 (35.8%)	17 (32.1%)	
	Internship	226 (23.6%)	13 (24.5%)	
Medical fields	Non-practitioner	120 (12.5%)	2 (3.8%)	0.057
	Practitioner	839 (87.5%)	51 (96.2%)	
Number of specialties of interest	One specialty	159 (16.6%)	7 (13.2%)	0.519
	More than One specialty	800 (83.4%)	46 (86.8%)	

*GPA* grade point average

* Number of participants who strongly agree and agree

**Table 3:** T test comparison in personality traits between neurosurgery and other specialties

	Mean (SD)		MD	*P* value
	Other	Neurosurgery		
Extraversion	3.29 (0.65)	3.39 (0.60)	-0.10	0.282
Agreeableness	3.79 (0.55)	3.70 (0.48)	0.08	0.271
Conscientiousness	3.76 (0.66)	3.69 (0.70)	0.07	0.426
Negative Emotionality	3.01 (0.73)	2.96 (0.83)	0.06	0.571
Open-Mindedness	3.61 (0.52)	3.88 (0.49)	-0.27	0.000

*SD* standard deviation, *MD* mean difference

**Table 4:** Binary logistic regression to assess for factors that influence neurosurgery specialty choice.

Factors	*P*-value	OR	95 CI	
			Lower	Upper
lifestyle or flexible work schedule	0.045	0.48	0.24	0.98
Workload or working hours	0.424	0.74	0.36	1.53
Absence of emergencies	0.410	0.74	0.37	1.50
Influence of GPA	0.004	2.45	1.32	4.54
Intellectual challenge	0.021	2.23	1.12	4.42
Length of training	0.513	0.78	0.37	1.62
Gender (female)	0.005	0.43	0.24	0.77
Medical Fields	0.198	2.59	0.60	11.05
Open-Mindedness	0.002	2.61	1.42	4.80

*OR* odds ratio, *CI* confidence interval

## Results

Table 1 displays the general characteristics of the study participants. Of the total participants, 39.3% were males, while 60.7% were females. Participants’ mean age was 23.3 years, with a standard deviation of 1.2 years. Regarding specialty choice, 5.2% of participants opted for neurosurgery.

The analysis identified significant differences between neurosurgery and other specialties in factors influencing specialty choice. Neurosurgery students were less likely to prioritize lifestyle or flexible work schedule (*p* = 0.000), workload (*p* = 0.001), absence of emergencies (*p* = 0.008), and length of training (*p* = 0.048) in addition they were more likely to prioritize the perceived influence of GPA (*p* = 0.005), intellectual challenge (*p* = 0.001) and being practitioner physician (*p* = 0.057) compared to students in other specialties. Additionally, a higher proportion of male students (58.5% males vs. 41.5% females) was observed in neurosurgery (*p* = 0.003). Results from t-test comparisons between neurosurgery and other specialties are shown in Table 3. Neurosurgery students displayed higher scores in Open-Mindedness (MD = -0.27, *p* < 0.001).

Regarding neurosurgery specialty choice on binary logistic regression (Table 4), factors that were associated with increased odds of selecting neurosurgery included a higher perceived influence of GPA (Odds Ratio [OR] = 2.45, 95% CI: 1.32–4.54, *p* = 0.004), perceiving the specialty as intellectually challenging (OR = 2.23, 95% CI: 1.12–4.42, *p* = 0.021), and being characterized as open-minded (OR = 2.61, 95% CI: 1.42–4.80, *p* = 0.002). Conversely, being female (OR = 0.43, 95% CI: 0.24–0.77, *p* = 0.005) and valuing lifestyle or flexible work schedule (OR = 0.48, 95% CI: 0.24–0.98, *p* = 0.045) were associated with significantly decreased odds of choosing neurosurgery. The logistic regression model was statistically significant, chi^2^ (9) = 55.49, *p* < 0.001, indicating that the included predictors significantly improved the model’s ability to distinguish between those who are interested in neurosurgery and those who are not. The model explained approximately 15.8% of the variance in career choice (Nagelkerke R^2^). A Hosmer and Lemeshow test yielded a non-significant result, chi^2^ (8) = 10.33, *p* = 0.243, suggesting that the model maintained a good fit with the observed data.

The gender-stratified analysis showed that certain factors remained significant for both genders, such as: lifestyle or flexible work schedule (males *p* = 0.011, females *p* = 0.011), intellectual challenge (males *p* = 0.006, females *p* = 0.035), and open-mindedness (males *p* = 0.012, females *p* = 0.007). Other factors were gender specific. Workload (*p* = 0.003) and GPA influence (*p* = 0.001) were significant only among males, whereas the absence of emergencies (*p* = 0.038) was significant only among females. Factors that lost statistical significance after stratification included the length of training (males *p* = 0.386, females *p* = 0.109) and practitioner physician status (males *p* = 0.116, females *p* = 0.271).

## Discussion

The primary aim of our study is to explore the factors and personality traits that impact the choice of medical students and interns to pursue a career in a neurosurgery specialty. Key findings revealed that factors associated with increased odds of selecting neurosurgery included a higher perceived influence of GPA, perceiving the specialty as intellectually challenging, and being characterized as open-minded. On the other hand, being a female and valuing lifestyle or flexible work schedule decreased these odds. We found that nearly 5.0% of participants were interested in neurosurgery, which is comparable to other studies. About 7% of the Nigerian medical students reported a willingness to choose neurosurgery as a career [18]. 

We found that participants who chose neurosurgery did not prioritize factors such as workload, absence of emergencies, and the length of training when deciding on their future specialty. Conversely, valuing lifestyle and work-life balance was significantly exhibited as a deterring factor from choosing a career in neurosurgery. Our findings are consistent with previous studies in which poor work-life balance related to the neurosurgery career was considered an important barrier against pursuing such a specialty. It was reported as one of the most common concerns among medical students planning to pursue a career in neurosurgery [3, 5, 6, 8, 9, 11, 19]. Work-life imbalance is a contributing factor to burnout, which has been reported in approximately 30%–67% of neurosurgery residents. Neurosurgery is recognized as a stressful and difficult profession owing to long working hours, difficult and very long training, unpredictable schedule, challenging training, inability to maintain work-life balance, the difficulty of neurological disorders, and poor patient outcomes [5, 7, 11, 20]. 

Interestingly, workload or working hours, absence of emergencies, and length of training were significant in the univariate analysis but not in the multivariate analysis. This suggests that their effects are explained by more encompassing factors in the regression model. In particular, workload and absence of emergencies appear to be captured by the broader construct of lifestyle or flexible work schedule. Students may not evaluate workload or emergency burden in isolation, but rather in terms of how these elements influence an overall ability to maintain a balanced schedule. In neurosurgery, the unpredictability and urgency of cases can disrupt flexibility, even when the overall case volume is not high. Also, students consider length of training in the context of lifestyle and long-term career satisfaction. An extended training pathway may be acceptable if it is balanced by a favorable lifestyle.

The influence of GPA and perceiving the specialty as intellectually challenging was significantly associated with an increased likelihood of choosing neurosurgery. Our finding that GPA influence predicts neurosurgery interest reflects perceived competitiveness rather than objective academic achievement. This suggests that students view neurosurgery as an academically elite field, and their decision to choose it is related to their perception of meeting these high academic standards. The existing literature provides mixed and inconclusive results regarding these factors. Similarly, Guadix et al. showed that interest in intellectually stimulating work attracts students toward neurosurgery [9]. Whereas, AlQahtani et al. revealed that perceiving neurosurgery specialty as intellectually challenging, including complicated neurosurgical disorders and difficulty in obtaining neurosurgery history and eliciting neurosurgery signs, was a deterring factor among the majority of medical students (97%) away from considering neurosurgery [11]. Differing initial interest levels, inadequate teaching and limited exposure to neurosurgery might affect the influence of the intellectual challenge on selecting it as a future career. The reason for this discrepancy may be due to the difference in participants’ interest in neurosurgery between the two studies. While the participants of Guadix et al. displayed a high level of interest in neurosurgery which increased even more after the course, the majority of AlQahtani et al. al participants showed a deviation of their interest away from neurosurgery since they were not considering it as their future specialty [9, 11]. Moreover, early exposure to neurosurgery and educational opportunities are crucial in shaping student interest [4, 10, 21–23]. However, some direct clinical rotation experiences, particularly in urban settings, can paradoxically decrease interest, possibly due to firsthand exposure to the demanding lifestyle [3, 24]. 

Among the five personality traits of the five-factor model, we discovered that only open-mindedness was significantly associated with an increased likelihood of selecting neurosurgery. The neurosurgical field requires innovative thinking and a willingness to tolerate uncertainty. A previous study reported that clinical neuroscience disciplines, including neurosurgery, neurology, and psychiatry, were distinguished by significantly higher open-mindedness scores than other specialties [25]. Furthermore, compared with clinicians in the other neuroscientific disciplines, neurosurgeons scored lower in neuroticism than neurologists, lower in agreeableness and neuroticism than psychiatrists, and higher in conscientiousness than psychiatrists [25]. Contrary to our initial expectations, no significant link was found between conscientiousness and interest in neurosurgery. This may reflect a mismatch between perceived and actual career demands, as students may view neurosurgery as requiring boldness, decisiveness and technical skill rather than high conscientiousness. Traits like honesty, motivation, willingness to learn, ability to problem solve and handle stress are also highlighted in literature as crucial for a neurosurgical career success [26]. 

Concerning gender differences, our study found that females were less inclined toward neurosurgery as their future specialty compared to males. This aligns with previous studies, which found that females are less inclined towards neurosurgery and have a higher attrition rate from residency programs [18, 27, 28]. In the Middle East, including Jordan, female representation in neurosurgery is low, though increasing resident numbers suggest future growth. In Jordan, as of 2021, there was only one female neurosurgeon and seven female residents [29]. This number is expected to rise, as nearly 40% (*n* = 22) of students interested in neurosurgery in our cohort were female. A research study conducted in the US showed that even though the proportion of females enrolling in neurosurgery residency programs has been increasing, this number is still out of proportion to the total number of females in medicine. Reasons include the challenging work nature, long working hours and its potential influence on the work-life balance and decreased family and personal time [11, 27, 30, 31]. Moreover, female medical students considered maternity/paternity leave more than their male counterparts [32]. Lack of proper exposure, cultural beliefs related to gender roles, insufficient mentorship (especially female mentors), and limited research opportunities are also significant factors. In a study among female medical students in India, 65.2% reported increased interest in pursuing neurosurgery if mentored by a female professor [28, 30]. 

The gender-stratified univariate analysis suggests that intellectual challenge and open-mindedness are universal drivers for Jordanian medical students; in addition, both female and male neurosurgery interested students placed less weight on lifestyle flexibility compared to those choosing other specialties. However, neurosurgery career choices are significantly shaped by gender-specific priorities. Female students drawn to neurosurgery were significantly less likely to prioritize the absence of emergencies than their peers who are interested in other specialties. Among males, those preferring neurosurgery were less likely to prioritize workload than those choosing other fields, indicating a psychological preparedness for the specialty’s demanding reputation. The loss of statistical significance for training length and practitioner physician status after stratification likely reflects a reduction in statistical power due to smaller subgroup sizes rather than a lack of relevance. These findings suggest the importance of gender sensitive mentorship strategies that address the distinct priorities of male and female medical students.

The importance of this study extends far beyond academic research. Understanding the factors and personality traits that influence the choice of specialization in neurosurgery has implications for medical education and healthcare delivery. Firstly, it can promote the development of medical curricula that support students interested in pursuing a career in neurosurgery. Additionally, if misconceptions or stigma surrounding neurosurgery persist, early exposure and mentorship programs can help eliminate these myths and direct students to pursue this challenging specialty. Moreover, policymakers will be better positioned to predict future workforce demands, guiding their investments in training programs to attract talents in neurosurgery, thus ensuring the quality and accessibility of neurosurgical care for diverse populations [2, 4, 21]. 

## Limitations

The study’s design limits interpretability and our ability to observe changes in personality traits over time and their longitudinal impact on career choices. Due to the cross-sectional nature of the study, which shows associations rather than causations as all variables are measured simultaneously at one point in time, the observed associations should not be interpreted as causal relationships, and reverse causality cannot be excluded. For instance, it cannot be determined whether open-mindedness predicts neurosurgery interest or vice versa. Prospective studies are needed to longitudinally track participants from preclinical years into residency matching outcomes and to examine how their traits and preferences evolve after starting residency training. Additionally, future studies should incorporate the perspectives of current residents across different specialties to provide a more comprehensive understanding of the factors influencing specialty selection.

The use of convenience sampling may introduce selection bias, potentially affecting the generalizability of our results, given participants’ varying levels of engagement with social media and online surveys. It is possible that the 5.2% interest in neurosurgery is an overestimation, as students with a specific interest in this demanding specialty or those with higher academic engagement (GPA influence) may have been more motivated to complete the survey. Moreover, our study’s low response rate (19.3%) suggests a potential non-response bias, as participants’ characteristics and motivations may differ from those of non-participants. It also indicates the relatively small sample size of the neurosurgery-interested participants subgroup (*n* = 53, 5.2%), which may have limited the statistical power and significance, and widened confidence intervals in regression analysis. Future studies could address these limitations through considering multi-country collaborations to increase the sample size and implementing targeted oversampling of students interested in neurosurgery. A multi-modal recruitment strategy should be employed, which includes combining social media recruitment with official university email lists, official academic channels, and in-class recruitment during clinical rotations to ensure a more diverse and representative participant pool.

Although English is the primary teaching language in Jordanian medical schools, the use of the BFI-2 in English may have posed comprehension challenges for some participants, as no validated Arabic version was available at the time of the study, potentially impacting response accuracy and causing measurement bias. We attempted to reduce this by providing the participants with the chance to contact the authors, who are proficient in both English and Arabic, for clarification regarding the questionnaire items. We recommend validating an Arabic version of the BFI-2 to minimize potential linguistic bias.

While we explored the perceived impact of GPA requirements on specialty choice, we did not include participants’ actual academic records in our analysis. Instead, we relied on their subjective perceptions of the GPA required for different specialties, which may not fully capture the influence of academic performance on career preferences. Therefore, our results should be interpreted as the impact of perceived academic prestige and competitiveness on career choice, which may not always correlate with a student’s objective academic performance. Thus, upcoming efforts should include objective academic performance data, such as self-reported GPA or verified university records (with appropriate ethical safeguards), to determine if actual academic success correlates with specialty interests.

## Conclusion

Choosing a medical specialty is a pivotal decision for medical students and young doctors. Our study focused on understanding how different factors and personality traits influence the choice to pursue neurosurgery among medical students and interns in Jordan. We found that factors such as GPA influence, perception of intellectual challenge, and personality traits like open-mindedness significantly influence the likelihood of selecting neurosurgery as a career. Conversely, concerns about lifestyle balance and gender disparities prevented many from choosing this demanding specialty. During career counseling, students interested in neurosurgery should be asked about their perspective on work-life balance. Those who demonstrate a willingness to prioritize intellectual fulfillment over lifestyle flexibility should be supported to pursue neurosurgery, as they may be better suited to thrive in its environment. However, while personality assessments have the potential to provide insight into career preference patterns and an applicant’s future behavior, they should not serve as a definitive selection criteria. It is important to exercise ethical caution regarding the use of psychometrics in residency recruitment, ensuring these tools are utilized for applicant self-reflection and guidance rather than deterministic selection. To address challenges in neurosurgery, there is a need for mentorship programs, particularly for women, and increased educational exposure to reduce misconceptions [10, 30]. Lastly, longitudinal studies could provide valuable insights into how these factors evolve throughout medical education and residency.

## Supplementary Information

Below is the link to the electronic supplementary material.

Supplementary Material 1

## Data Availability

The datasets generated and/or analyzed during the current study are available from the corresponding author upon reasonable request.

## Abbreviations

BFI-2: Big Five Inventory–2
CI: Confidence Interval
GPA: Grade Point Average
IRB: Institutional Review Board
OR: Odds Ratio
SD: Standard Deviation
