# Supplementary material for: Exploring the factors and personality traits influencing neurosurgery career choice in Jordan
Source: Langenbecks Arch Surg. 2026 May 12;411(1):183. doi: 10.1007/s00423-026-04068-5 (PMC13337725; doi:10.1007/s00423-026-04068-5)
Supplement: Supplementary file 1 — Supplementary Material 1 [file 423_2026_4068_MOESM1_ESM.docx]

# Appendix

**Appendix 1: Gender-Stratified Analysis of the differences between those aspiring neurosurgery and those aspiring other specialties regarding factors influencing students’ choice of future specialty.**

| **Factor** | **Gender** | **Level** | **Other Specialty**** | **Neurosurgery**** | **P-value** |
| --- | --- | --- | --- | --- | --- |
| Anticipated income | Male | Agree* | 252 (68.7%) | 22 (71.0%) | 0.790 |
|  | Female | Agree | 374 (63.2%) | 10 (45.5%) | 0.102 |
| Lifestyle or flexible work schedule | Male | Agree | 268 (73.0%) | 16 (51.6%) | 0.011 |
|  | Female | Agree | 440 (74.3%) | 11 (50.0%) | 0.011 |
| Workload or working hours | Male | Agree | 209 (56.9%) | 9 (29.0%) | 0.003 |
|  | Female | Agree | 389 (65.7%) | 12 (54.5%) | 0.280 |
| Absence of emergencies | Male | Agree | 145 (39.5%) | 8 (25.8%) | 0.132 |
|  | Female | Agree | 267 (45.1%) | 5 (22.7%) | 0.038 |
| Professional prestige | Male | Agree | 221 (60.2%) | 20 (64.5%) | 0.638 |
|  | Female | Agree | 291 (49.2%) | 10 (45.5%) | 0.733 |
| Specialty is well-respected | Male | Agree | 244 (66.5%) | 23 (74.2%) | 0.380 |
|  | Female | Agree | 359 (60.6%) | 13 (59.1%) | 0.884 |
| Occupational satisfaction | Male | Agree | 309 (84.2%) | 27 (87.1%) | 0.669 |
|  | Female | Agree | 515 (87.0%) | 20 (90.9%) | 0.590 |
| Personal interests | Male | Agree | 318 (86.6%) | 25 (80.6%) | 0.352 |
|  | Female | Agree | 537 (90.7%) | 21 (95.5%) | 0.448 |
| Academic interests | Male | Agree | 237 (64.6%) | 22 (71.0%) | 0.474 |
|  | Female | Agree | 436 (73.6%) | 15 (68.2%) | 0.569 |
| Personal skills or competencies in the specialty | Male | Agree | 291 (79.3%) | 26 (83.9%) | 0.543 |
|  | Female | Agree | 462 (78.0%) | 15 (68.2%) | 0.275 |
| Influence of media | Male | Agree | 84 (22.9%) | 9 (29.0%) | 0.438 |
|  | Female | Agree | 133 (22.5%) | 6 (27.3%) | 0.597 |
| Influence of family members | Male | Agree | 111 (30.2%) | 12 (38.7%) | 0.327 |
|  | Female | Agree | 183 (30.9%) | 7 (31.8%) | 0.928 |
| Influence of a professor or resident during medical school | Male | Agree | 175 (47.7%) | 15 (48.4%) | 0.940 |
|  | Female | Agree | 245 (41.4%) | 9 (40.9%) | 0.964 |
| Role model | Male | Agree | 145 (39.5%) | 15 (48.4%) | 0.333 |
|  | Female | Agree | 220 (37.2%) | 6 (27.3%) | 0.345 |
| Influence of having a doctor in the family | Male | Agree | 91 (24.8%) | 12 (38.7%) | 0.101 |
|  | Female | Agree | 129 (21.8%) | 5 (22.7%) | 0.917 |
| Influence of GPA | Male | Agree | 63 (17.2%) | 13 (41.9%) | 0.001 |
|  | Female | Agree | 142 (24.0%) | 7 (31.8%) | 0.400 |
| Career opportunities (availability) | Male | Agree | 213 (58.0%) | 21 (67.7%) | 0.292 |
|  | Female | Agree | 372 (62.8%) | 10 (45.5%) | 0.101 |
| Desire to specialize abroad | Male | Agree | 247 (67.3%) | 22 (71.0%) | 0.675 |
|  | Female | Agree | 305 (51.5%) | 14 (63.6%) | 0.264 |
| Intellectual challenge | Male | Agree | 191 (52.0%) | 24 (77.4%) | 0.006 |
|  | Female | Agree | 323 (54.6%) | 17 (77.3%) | 0.035 |
| Long-term relationship with patients | Male | Agree | 131 (35.7%) | 12 (38.7%) | 0.737 |
|  | Female | Agree | 234 (39.5%) | 6 (27.3%) | 0.247 |
| Gender distribution in the specialty | Male | Agree | 80 (21.8%) | 6 (19.4%) | 0.751 |
|  | Female | Agree | 161 (27.2%) | 8 (36.4%) | 0.344 |
| Peer group choices | Male | Agree | 58 (15.8%) | 6 (19.4%) | 0.605 |
|  | Female | Agree | 76 (12.8%) | 3 (13.6%) | 0.913 |
| Personal experience with a disease | Male | Agree | 101 (27.5%) | 9 (29.0%) | 0.857 |
|  | Female | Agree | 180 (30.4%) | 6 (27.3%) | 0.754 |
| Diversity of patients | Male | Agree | 149 (40.6%) | 14 (45.2%) | 0.620 |
|  | Female | Agree | 243 (41.0%) | 11 (50.0%) | 0.402 |
| Lack of competitiveness | Male | Agree | 118 (32.2%) | 12 (38.7%) | 0.455 |
|  | Female | Agree | 194 (32.8%) | 3 (13.6%) | 0.109 |
| Length of training | Male | Agree | 110 (30.0%) | 7 (22.6%) | 0.386 |
|  | Female | Agree | 235 (39.7%) | 5 (22.7%) | 0.109 |
| Academic year | Male | Fifth | 153 (41.7%) | 12 (38.7%) | 0.801 |
|  |  | Sixth | 127 (34.6%) | 10 (32.3%) |  |
|  |  | Internship | 87 (23.7%) | 9 (29.0%) |  |
|  | Female | Fifth | 237 (40.0%) | 11 (50.0%) | 0.636 |
|  |  | Sixth | 216 (36.5%) | 7 (31.8%) |  |
|  |  | Internship | 139 (23.5%) | 4 (18.2%) |  |
| Medical fields | Male | Non-practitioner | 47 (12.8%) | 1 (3.2%) | 0.116 |
|  |  | Practitioner | 320 (87.2%) | 30 (96.8%) |  |
|  | Female | Non-practitioner | 73 (12.3%) | 1 (4.5%) | 0.271 |
|  |  | Practitioner | 519 (87.7%) | 21 (95.5%) |  |
| Number of specialties | Male | One specialty | 65 (17.7%) | 5 (16.1%) | 0.824 |
|  |  | More than One specialty | 302 (82.3%) | 26 (83.9%) |  |
|  | Female | One specialty | 94 (15.9%) | 2 (9.1%) | 0.389 |
|  |  | More than One specialty | 498 (84.1%) | 20 (90.9%) |  |

GPA: Grade Point Average.
* Number of participants who strongly agree and agree. ** Number of males and females interested in other specialties are 367 and 592, respectively. Number of males and females interested in neurosurgery are 31 and 22, respectively.

**Appendix 2: Gender-Stratified Analysis to compare personality traits between neurosurgery and other specialties.**

|  | **Mean (SD)** | | **MD** | **P value** |
| --- | --- | --- | --- | --- |
|  | **Other** | **Neurosurgery** |  |  |
| **Male** |  |  |  |  |
| Extraversion | 3.35 (0.63) | 3.33 (0.64) | 0.02 | 0.869 |
| Agreeableness | 3.62 (0.56) | 3.71 (0.46) | -0.09 | 0.398 |
| Conscientiousness | 3.69 (0.65) | 3.72 (0.70) | -0.03 | 0.811 |
| Negative Emotionality | 2.88 (0.73) | 2.87 (0.87) | 0.01 | 0.920 |
| Open-Mindedness | 3.61 (0.53) | 3.86 (0.49) | -0.25 | 0.012 |
| **Female** |  |  |  |  |
| Extraversion | 3.26 (0.66) | 3.48 (0.54) | -0.22 | 0.118 |
| Agreeableness | 3.89 (0.52) | 3.70 (0.52) | 0.20 | 0.082 |
| Conscientiousness | 3.81 (0.66) | 3.64 (0.71) | 0.16 | 0.257 |
| Negative Emotionality | 3.09 (0.71) | 3.08 (0.78) | 0.02 | 0.902 |
| Open-Mindedness | 3.61 (0.50) | 3.91 (0.49) | -0.30 | 0.007 |

SD; standard deviation, MD; mean difference
